# Cyclophilin A Restricts Influenza A Virus Replication through Degradation of the M1 Protein

**DOI:** 10.1371/journal.pone.0031063

**Published:** 2012-02-08

**Authors:** Xiaoling Liu, Zhendong Zhao, Chongfeng Xu, Lei Sun, Jilong Chen, Lianfeng Zhang, Wenjun Liu

**Affiliations:** 1 Center for Molecular Virology, CAS Key Laboratory of Pathogenic Microbiology and Immunology, Institute of Microbiology, Chinese Academy of Sciences, Beijing, China; 2 Graduate University of Chinese Academy of Sciences, Beijing, China; 3 China-Japan Joint Laboratory of Molecular Immunology and Molecular Microbiology, Institute of Microbiology, Chinese Academy of Sciences, Beijing, China; 4 Key Laboratory of Human Disease Comparative Medicine, Ministry of Health, Institute of Laboratory Animal Science, Chinese Academy of Medical Sciences and Comparative Medical Center, Peking Union Medical College, Beijing, China; University of Cambridge, United Kingdom

## Abstract

Cyclophilin A (CypA) is a typical member of the cyclophilin family of peptidyl-prolyl isomerases and is involved in the replication of several viruses. Previous studies indicate that CypA interacts with influenza virus M1 protein and impairs the early stage of the viral replication. To further understand the molecular mechanism by which CypA impairs influenza virus replication, a 293T cell line depleted for endogenous CypA was established. The results indicated that CypA inhibited the initiation of virus replication. In addition, the infectivity of influenza virus increased in the absence of CypA. Further studies indicated that CypA had no effect on the stages of virus genome replication or transcription and also did not impair the nuclear export of the viral mRNA. However, CypA decreased the viral protein level. Additional studies indicated that CypA enhanced the degradation of M1 through the ubiquitin/proteasome-dependent pathway. Our results suggest that CypA restricts influenza virus replication through accelerating degradation of the M1 protein.

## Introduction

Influenza virus is an enveloped negative-sense RNA virus that causes major public health problems worldwide. The matrix protein (M1) is the most abundant protein in the viral particle and forms the bridge between the viral envelope and the core. M1 protein is a multifunctional protein in the influenza virus life cycle including uncoating, transcription, the nuclear export of vRNP, assembly and budding. Several host cell factors have been determined possibly to be required for regulation of influenza virus replication through interacting with M1 at different stages of infection [1], [2], [3], [4]. In the previous study, Cyclophilin A (CypA) was identified to interact with influenza virus M1 protein and impair the early stage of the viral replication [5]. In the present study, CypA might regulate the viral protein stability at the post-translation level of influenza virus life cycle.

Post-translational modification of proteins by ubiquitin is a key regulatory event in many cellular activities, such as signal transduction, transcription, nuclear transport, membrane protein trafficking, autophagy, and immune responses [6]. Previous studies suggest an important involvement of the ubiquitin proteasome system (UPS) in the influenza virus infection. For example, the ubiquitin-vacuolar protein sorting system is required during entry of influenza virus into cells [7]. Further studies indicate that inhibition of the UPS affects influenza virus infection at a post-fusion step [8]. Influenza virus inhibits host interferon response through NS1 targeting the ubiquitin ligase TRIM25 [9]. Influenza A virus RNA replication was regulated through the ubiquitination and deubiquitination of NP protein [10]. However, the ubiquitination of influenza A virus M1 protein is still unknown.

CypA is a member of the immunophilin superfamily that has peptidyl-prolyl cis-trans isomerase activity. Several lines of evidence implicate that CypA can aid protein folding due to its isomerase activity, and it is also active in cell signaling [11], [12], [13], [14]. In addition, CypA is involved in the life cycles of several viruses, such as human immunodeficiency virus type 1 (HIV-1), influenza virus, vesicular stomatitis virus (VSV), vaccinia virus (VV), hepatitis C virus (HCV) and hepatitis B virus (HBV) [5], [15], [16], [17], [18], [19], [20], [21]. Another member of the immunophilin superfamily, Pin1, has been reported to be involved in the UPS. Pin1 stabilizes the human T-cell leukemia virus type 1 (HTLV-1) Tax oncoprotein and promotes malignant transformation [22]. Pin1 regulates NF-κB signaling through the UPS [23]. In the reports related to influenza virus, CypA was shown to be in the core of the influenza virion [24] and was up-regulated upon infection by avian H9N2 influenza virus in a human gastric carcinoma cell line (AGS) [25]. Furthermore, both human and chicken CypA specifically interacted with the M1 protein and suppressed the viral replication. In addition, the isomerase activity of CypA is not necessary for viral replication [5], [26], but the precise functions and roles of CypA in the influenza virus life cycle have not yet been elucidated. Thus, it is of interest to further understand how CypA participates in viral replication.

A cell line depleted of endogenous CypA would be a useful model to understand the precise functions of CypA in the influenza virus life cycle. Therefore, in the present study, a stable RNAi 293T cell line with maximally decreased CypA expression (293T/CypA−) was established as described in [27]. The replication of influenza A virus in the 293T/CypA− and 293T (i.e., 293T/CypA+) cell lines was characterized to further determine the effects of CypA on virus replication. The present data indicated that CypA inhibited influenza virus replication through accelerating degradation of the M1 protein.

## Results

### CypA inhibited influenza A virus replication

**Figure 1:**
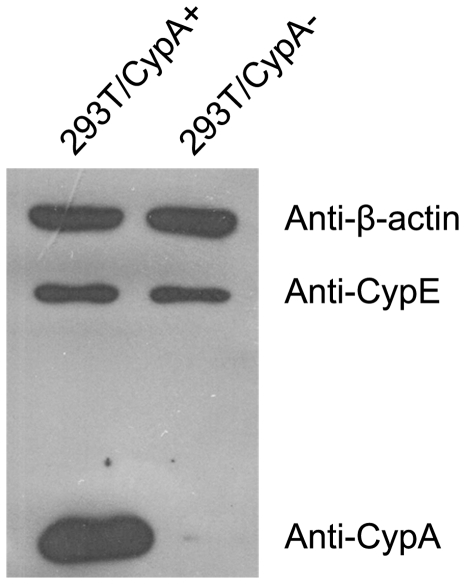
Characterization of the 293T/CypA− cell lines. Western blot of total cell lysates from the 293T/CypA+ and 293T/CypA− cell lines. The samples were analyzed by immunoblotting with polyclonal anti-CypA and anti-CypE antibodies. β-actin was used as a control.

**Figure 2:**
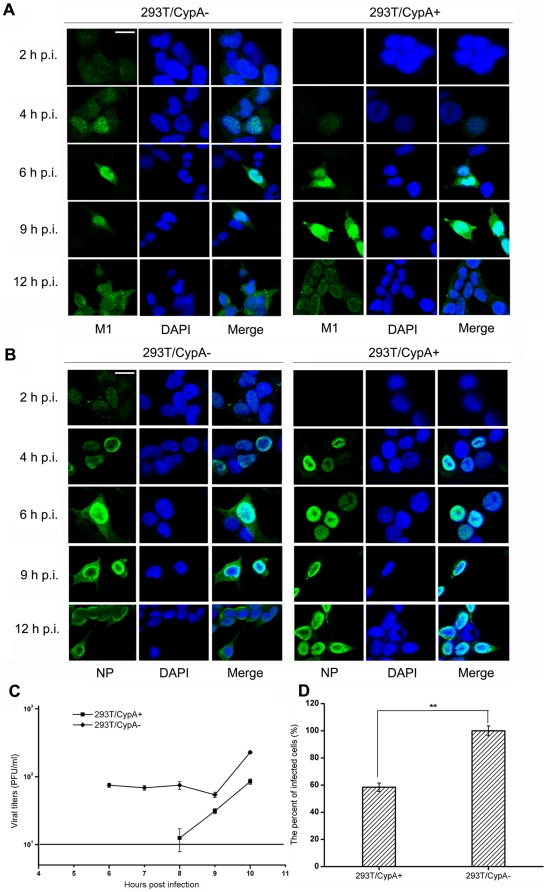
CypA inhibited influenza A virus replication. Localization of the M1 (A) and NP (B) proteins in WSN-infected 293T/CypA+ and 293T/CypA− cells. 293T/CypA+ and 293T/CypA− cells were infected with WSN at an MOI of 1. At 2, 4, 6, 9, and 12 h post-infection, cells were fixed, permeabilized and stained with anti-M1 (green in A), anti-NP (green in B) and DAPI (blue). (Scale bars = 10 µm). C: One-step growth curves of WSN in the 293T/CypA+ and 293T/CypA− cell lines. The cells were counted, cultivated, and infected at an MOI of 1. Quantitation of infectious progeny viral particles generated from infected cells by standard plaque assays. Data are presented as the standard error of the mean (SEM) from six independent experiments. The limit of detection of the plaque assays was 10 PFU/ml was shown by the straight line. D: Different infectivities of influenza virus in the 293T/CypA+ and 293T/CypA− cell lines. The cells were counted, cultivated, and infected (MOI = 1). At 4 h post-infection, the cells were fixed and stained for NP protein (green). The percent of green cells was calculated, and this reflected the infectivity of the influenza virus. Data are presented as the mean plusminus standard deviation (±SD) from three independent experiments. Significant differences (P <0.01, t-test) are indicated by two asterisks.

To better evaluate the function of CypA during viral infection, a 293T cell line depleted of CypA expression (293T/CypA−) was established as described in the materials and methods. Several clonal populations of GFP-expressing cells were obtained and analyzed for CypA depletion by characterizing the relative amounts of CypA and cyclophilin E (CypE). The expression level of CypA in the 293T/CypA− cell line was not detectable by western blotting, but the amount of CypE protein (another member of the Cyp family) in both cell lines was similar, as shown in Figure 1. In addition, except for the expression level of CypA, no significant differences in cell morphology and cell cycle were observed between the two cell lines (data not shown). We then examined the intracellular distribution patterns of the M1 and NP proteins in A/WSN/33-infected 293T/CypA+ and 293T/CypA− cell lines (MOI = 1) by indirect immunofluorescence assays (Figure 2A, B). At the early stage (2 h post-infection), M1 was not yet visualized in 293T/CypA+ cells, but it could be visualized in 293T/CypA− cells. At 4 h post-infection, M1 was detected to be evenly localized in the nucleus and cytoplasm of 293T/CypA+ cells and higher levels of M1 were found to be localized in the nucleus of 293T/CypA− cells. At the middle stage (6 h post-infection) of infection, M1 was mainly localized in the nucleus in both cell lines, and at 9 h post-infection, similar results were observed in both cell lines. However, at the late stage of infection (12 h post-infection), M1 was dispersed throughout the entire cell in the 293T/CypA− cell line. In contrast, M1 was mainly distributed in the nucleus and nuclear periphery in 293T/CypA+ cells (Figure 2A). A similar phenomenon was observed for the location of NP (Figure 2B). At 2 h post-infection, NP was first detected in 293T/CypA− but not in 293T/CypA+ cells. At 6 and 9 h post-infection, NP was located in the nucleus of 293T/CypA+ cells, but in 293T/CypA− cells, NP was found in both the nucleus and cytoplasm. Furthermore, at 12 h post-infection, NP had mainly translocated from the nucleus into the cytoplasm in 293T/CypA− cells but was still largely located in the nucleus of 293T/CypA+ cells (Figure 2B). The differing locations of the two viral proteins showed that influenza A virus replication appeared to be faster in 293T/CypA− than in 293T/CypA+ cell line.

To determine the impact of CypA on influenza virus replication, the one-step growth curves of influenza A/WSN/33 were examined in the 293T/CypA+ and 293T/CypA− cell lines (Figure 2C). These cell lines were infected (MOI = 5), and the supernatant was titrated by plaque assay at every hour post infection. Virions in the supernatant of 293T/CypA− cells were first detected at 6 h post-infection, but in the supernatant of 293T/CypA+ cells, virions were not detected until 8 h post-infection. The results indicated that the first life cycle of influenza virus in 293T/CypA− was ∼2 h shorter than in the 293T/CypA+ cell line.

To determine the infectivity of influenza virus in both 293T/CypA+ and 293T/CypA− cell lines, indirect immunofluorescence assays were performed to detect NP protein at 4 h post-infection (MOI = 1) in both cell lines (Figure 2D). An analysis of 300 cells indicated that the percent of infected cells of the 293T/CypA+ cell line was only 58.4% of that in the 293T/CypA− cell line. The results indicated that the amount of CypA affected the viral infection. Thus, the results of both assays above indicated that host factor CypA inhibited influenza A virus replication.

### CypA regulated influenza virus replication at the post-transcription level

**Figure 3:**
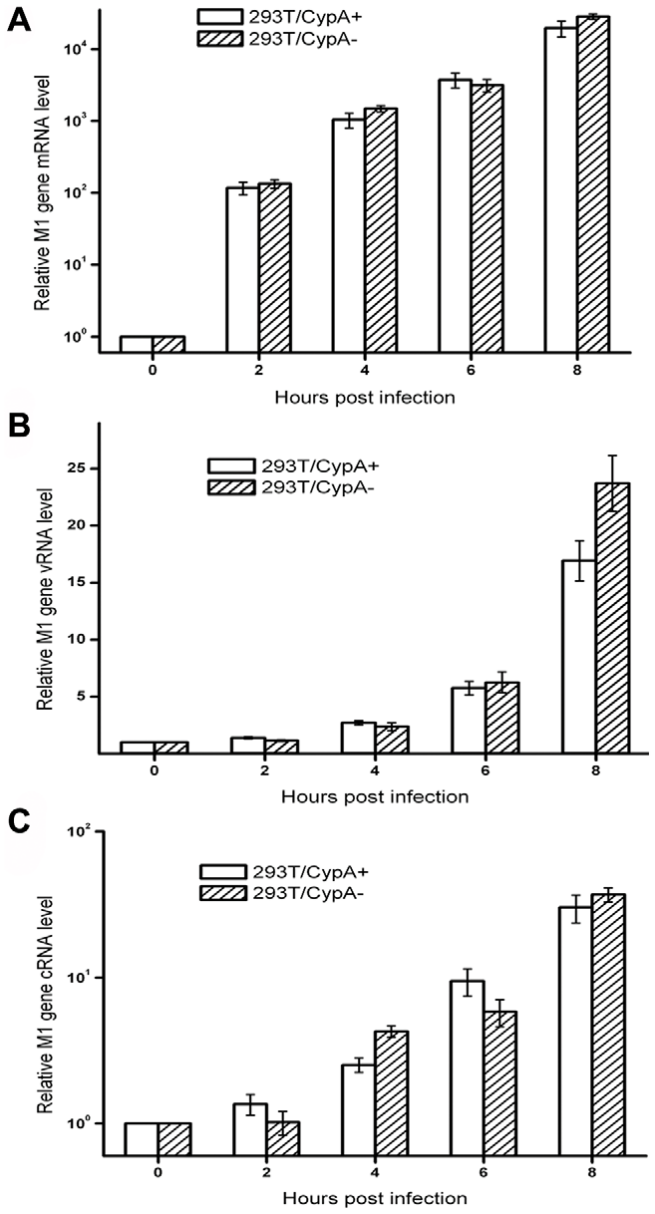
CypA suppressed influenza virus replication at the post-transcription level. Relative quantitation of M1 mRNA (A), vRNA (B), and cRNA (C) in cells infected with A/WSN/33 at different time points post-infection. As described in the experimental procedures, different primers were used for the cDNA synthesis of mRNA, vRNA, and cRNA. Comparison of viral RNA levels from influenza A M1 genes in the presence or absence of CypA. The specificities of the amplified products were all confirmed by melting curve analysis. In all of the PCR assays, GAPDH was used as an internal control. Data are means ± SD of three separate experiments.

To identify the exact stage of viral replication regulated by CypA, real-time PCR assays were performed to detect M1 mRNA, vRNA, and cRNA levels upon influenza virus infection in the 293T/CypA+ and 293T/CypA− cell lines. The results demonstrated that the M1 mRNA level was similar in both 293T/CypA+ and 293T/CypA− cells (Figure 3A), and there was no significant differences at the vRNA or cRNA level in both cell lines (Figure 3B, C). These results suggested that CypA had no effect on the virus genome replication and transcription of influenza virus and instead regulated the viral life cycle at the post-transcription level.

### CypA did not impair the nuclear export of viral mRNA

**Figure 4:**
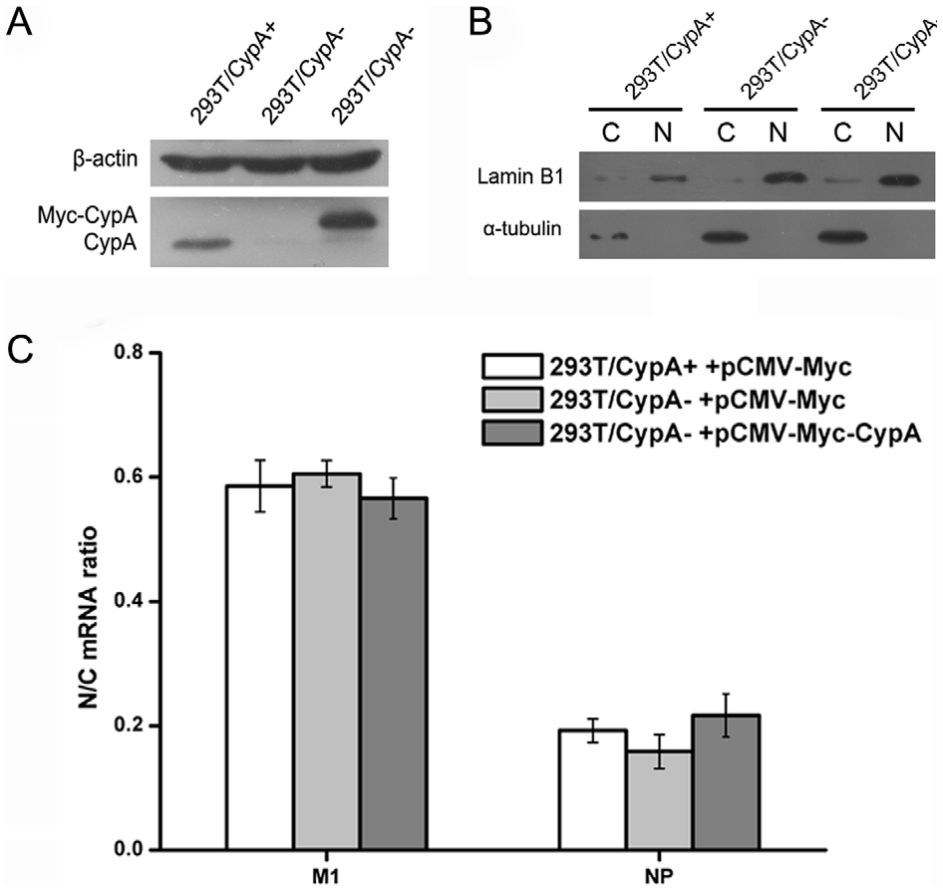
CypA did not impair the nuclear export of influenza virus mRNA. The 293T/CypA+, 293T/CypA−, and CypA re-expression 293T/CypA− cell lines were infected with A/WSN/33 (MOI = 0.1). At 4 h post-infection, the CypA expression level was analyzed by western blotting using a polyclonal anti-CypA antibody in the three cell lines. β-actin was used as a control (A). Nuclear (N) and cytoplasmic (C) components were isolated from the various cell lines. One tenth of the components were used for Western blot by anti-α-tubulin and anti-lamin B1 (B). The rest of the components were used for the RNA extraction and real time PCR (C). M1 and NP mRNA levels were quantified by real-time RT-PCR using gene-specific primers. GAPDH was quantified as an internal control. Data are means ± SD of three separate experiments.

As was shown, CypA did not affect the transcription and genome replication of influenza virus. To determine if CypA has an effect on viral mRNA nuclear export, we compared the nuclear and cytoplasmic abundance of M1 and NP mRNA upon influenza virus infection at 4 h post-infection in the 293T/CypA+ and 293T/CypA− cell lines. In addition, we also rescued the expression of CypA in the 293T/CypA− cell line and then analyzed the nuclear and cytoplasmic abundance of M1 and NP mRNA upon influenza virus infection. First, we determined the CypA protein level in the 293T/CypA+, 293T/CypA− and “CypA re-expression” 293T/CypA− cell lines (Figure 4A). Then nuclear and cytoplasmic fractions were isolated and analyzed by Western blotting for lamin B1 and α-tubulin (Figure 4B).The results showed that the nuclear/cytoplasmic fractions were isolated well. Further studies indicated that the ratio of nuclear/cytoplasm M1 mRNA was similar in all three lines (Figure 4C). In addition, we obtained similar results for the pattern of NP mRNA (Figure 4C). These results suggested that the presence of CypA in the cell had no effect on the nuclear export of viral mRNA.

### CypA affected the stability of the M1 protein

**Figure 5:**
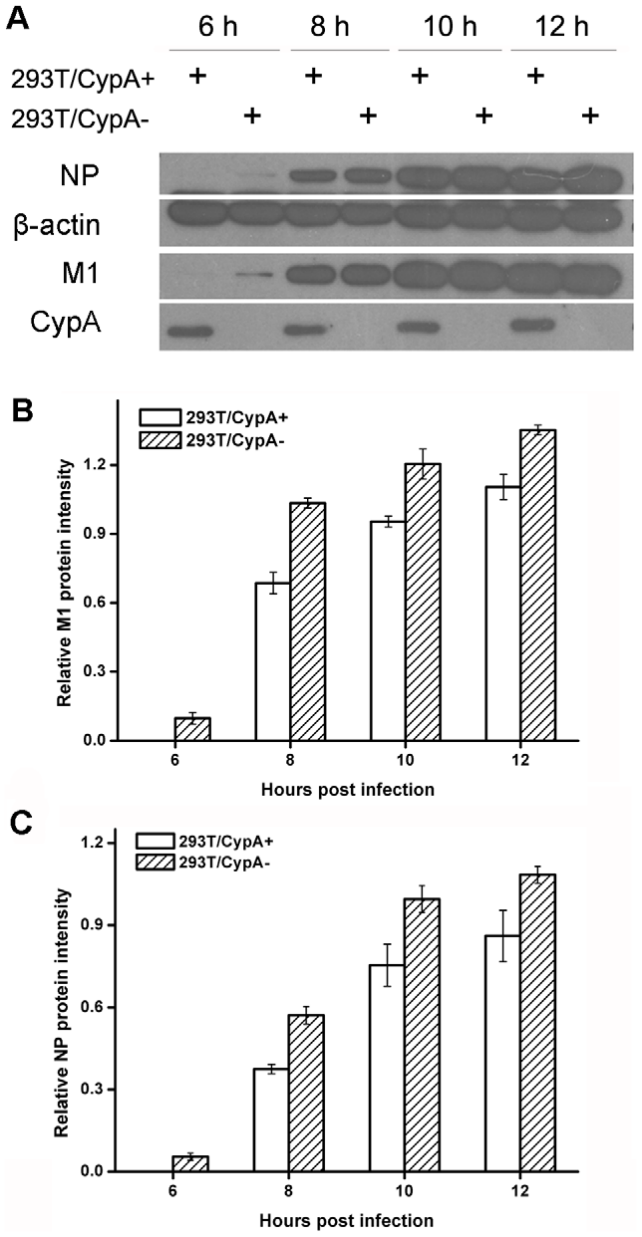
CypA inhibited the replication of influenza virus at the viral protein level. The 293T/CypA+ and 293T/CypA− cell lines were infected with influenza A virus (MOI = 0.1) and lysed in lysis buffer at 6, 8, 10, and 12 h post-infection The samples were analyzed by western blotting with a polyclonal anti-CypA antibody, monoclonal anti-M1 antibody, and polyclonal anti-NP antibody; β-actin was used as a control (A). Relative expression levels of M1 (B) and NP (C) were calculated by quantifying the results shown in panel (A). Data are means ± SD of three separate experiments.

As demonstrated above, CypA had no effect on influenza virus genome transcription, replication, or on the nuclear export of viral mRNA. Thus, we investigate if CypA impairs the viral protein level upon infection. The protein levels of M1 after infection were detected at various time points in both cell lines (Figure 5A). At 6 h post-infection, M1 was detected in 293T/CypA− but not in 293T/CypA+ cells. At 8 h post-infection, the expression of M1 in 293T/CypA− cells was 50% higher than that in 293T/CypA+ cells, and at 10 h post-infection, the M1 protein level in 293T/CypA− was 32% higher than in 293T/CypA+ cells. At 12 h post-infection in 293T/CypA− cells, the M1 protein level was only 26% greater than in 293T/CypA+ cells (Figure 5B). A similar phenomenon was observed for the NP protein level (Figure 5C). Thus, the above results suggested that one function of CypA was reducing the viral proteins content and inhibiting the viral replication at protein level.

**Figure 6:**
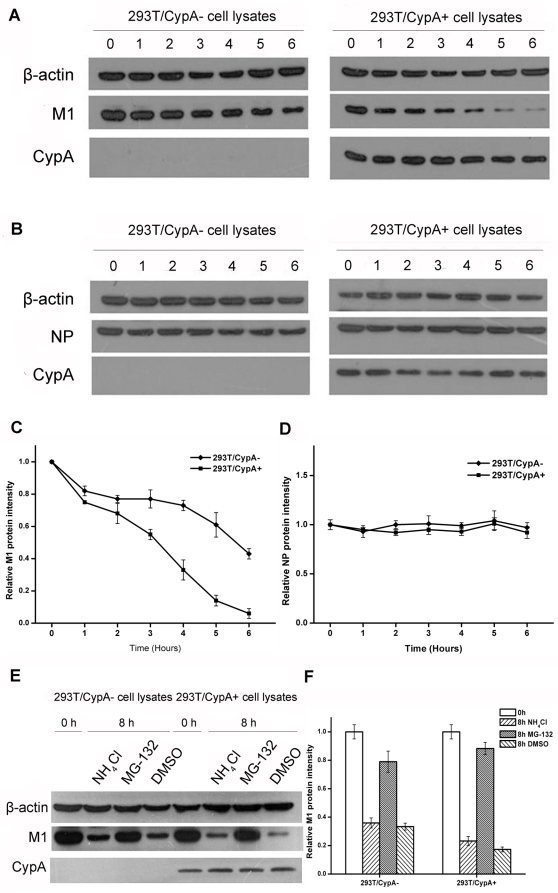
CypA accelerated the degradation of M1 protein through the proteasome-dependent pathway. Both 293T/CypA− and 293T/CypA+ cell lines were transfected with pcDNA3-FLAG-M1 (A) or pcDNA3-FLAG-NP (B), respectively. Cells were then treated with CHX (100 µg/ml) 24 h post-transfection, and these cells were lysed at different time points. The samples were analyzed by western blotting with anti-β-actin, anti-M1, anti-NP, and anti-CypA antibodies. Relative expression levels of M1 (C) and NP (D) were quantified. Data are presented as the ±SD from three separate experiments. (E) Protein degradation of M1 protein was blocked by the proteasome inhibitor. The 293T/CypA− and 293T/CypA+ cell lines were transfected with pcDNA3-FLAG-M1. CHX was then added 24 h post-transfection, and DMSO, lysosome inhibitor (NH_4_Cl), and proteasome inhibitor (MG-132) were added for 8 h. The cell lysates were harvested and analyzed by western blotting with anti-M1, anti-CypA, and anti-β-actin antibodies. The relative protein level of M1 was calculated by quantifying the results shown in panel (F). Data are presented as the ±SD from three separate experiments.

To analyze whether CypA influences viral protein stability, protein degradation assays were performed. First, both 293T/CypA+ and 293T/CypA− cell lines were transiently transfected with pcDNA3-FLAG-M1 or pcDNA3-FLAG-NP, respectively. Cycloheximide (CHX) was then added 24 h post-transfection. Cells were collected at 0, 1, 2, 3, 4, 5, and 6 h post-treatment with CHX, and western blot analyses were performed for M1 or NP (Figure 6A, B). The quantitative analyses demonstrated that the half-life of M1 protein in 293T/CypA+ cell was nearly 2 h but almost 6 h in 293T/CypA− cells (Figure 6C). This experiment revealed that the protein stability of M1 was significantly reduced in 293T/CypA+ compared to 293T/CypA− cells, indicating that CypA decreased the protein stability of M1. Meanwhile, the protein stability of NP was similar in both 293T/CypA+ and 293T/CypA− cells 6 h after treatment (Figure 6B, D).

There are two different pathways, proteasome- and lysosome-dependent, that are principally responsible for protein degradation in cells. To address which pathway mediated the degradation of M1, we performed parallel experiments using either the proteasome inhibitor MG-132 or the lysosome inhibitor NH_4_Cl. Treatment with MG-132 significantly inhibited the degradation of M1 in both cell lines, whereas the NH_4_Cl treatment did not inhibit M1 degradation (Figure 6E). In addition, the M1 protein level was different in MG-132-treated cell lines compared to controls. As shown in Figure 6F, the M1 protein level in MG-132-treated 293T/CypA+ cells was ∼5-fold greater than in DMSO-treated 293T/CypA+ cells. Similarly, in the 293T/CypA− cell line, the M1 protein level in MG-132-treated cells was 2-fold greater than the DMSO control. These results indicated that the degradation of M1 in both cell lines occurs via the proteasome-dependent pathway, and CypA specifically accelerates the degradation of M1 protein through this pathway.

**Figure 7:**
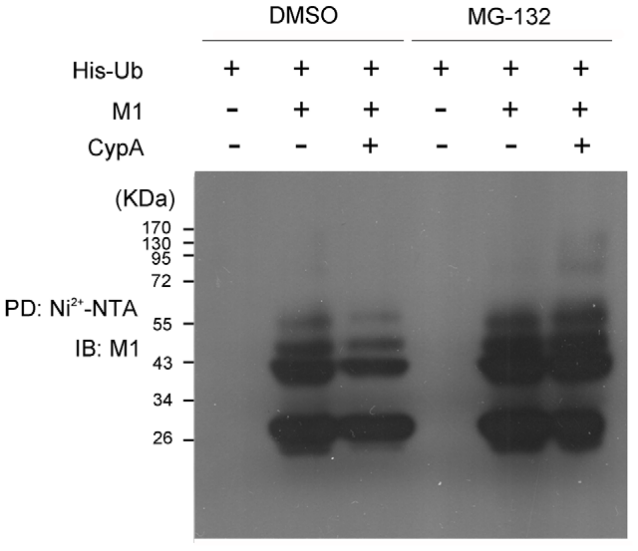
In vivo ubiquitination assay suggested that CypA increased the ubiquitination of the M1 protein. 293T/CypA− cells were transfected with expression constructs for Myc-tagged CypA, FLAG-tagged full-length M1, and His-tagged ubiquitin. Forty hours post-transfection, the proteasome inhibitor MG-132 (dissolved in DMSO) was added to the cells for 4 h at a final concentration of 10 µM. Cell extracts were then immunoprecipitated with Ni2+-NTA beads. The eluted proteins were analyzed by western blotting using an anti-FLAG monoclonal antibody.

To further determine if CypA affects the ubiquitination status of M1, an in vivo ubiquitination assay was performed in CypA−, M1- and His-ubiquitin-co-expressing cell lysates. Surprisingly, CypA decreased both the level of non-ubiquitinated M1 and the level of poly-ubiquitinated M1 species (Figure 7). When the proteasomal function was inhibited by MG-132, non- and poly-ubiquitinated M1 species accumulated to higher levels than in the control. Furthermore, CypA over-expression increased the non- and poly-ubiquitinated M1 species.

## Discussion

CypA, as an intrinsic host defense factor, regulated influenza virus replication. These phenomena were observed including the difference of location of viral proteins and the reduction of the infectivity of the virus, which reflected the state of the viral proteins. To dig the accurate mechanism of the effect of CypA in the life cycle of influenza virus, we detected the viral RNA level, the location of viral mRNA, the viral proteins expression and the stability of these viral proteins. After longitudinal comparing these results, we concluded that CypA inhibited influenza virus replication at the post-transcription level and maybe through accelerating the degradation of the M1 protein.

In a previous study, CypA was shown to interact with M1 and suppressed influenza virus replication [5]. Immunocytological assays indicated that over-expressed CypA may prevent M1 protein from entering the nucleus at 4 h post-infection [5]. Further studies may provide another explanation of the results that CypA accelerated the degradation of the M1 protein and so we detected it in the infected cells a little bit late. In addition, M1 protein was prone to degradation in the presence of CypA and then the timing of the influenza virus replication would be delayed. Therefore, at different time points post influenza virus infection, both M1 and NP protein levels were lower in 293T/CypA+ than in 293T/CypA− infected cell lysates (Figure 5).

In the present study, we found that CypA did not affect influenza virus transcription and virus genomes replication or the nuclear export of the viral mRNA but influenced the viral protein level. So we concluded that CypA inhibits influenza virus replication at the translational level through impairing the viral protein synthesis or post-translation level by regulating the stability of the M1 protein. Our data suggested that CypA accelerated the degradation of M1 protein (Figure 6). But we could not rule out the other possibility that CypA might inhibit the viral replication through impairing the viral proteins translation. Translational control of influenza virus is exerted at several steps in the viral life cycle. Thus, the virus has evolved strategies to take advantage of its requirement for cap-dependent translation initiation, as well as the recruitment of host cell proteins to aid in the translation of viral mRNA [28]. However, the host cell has also evolved to counteract the virus' mechanisms to bolster viral protein synthesis. For example, since viral translation is dependent upon functional eIF2α for translation initiation, viruses have developed strategies to prevent or reverse PKR-dependent eIF2α phosphorylation [29]. Influenza virus nonstructural (NS1) protein could interfere with dsRNA binding to PKR, thus preventing PKR dimerization and autophosphorylation [30]. So whether or not CypA regulates influenza viral protein translation need further study.

The amount of endogenous CypA in cells plays an important role in many virus infections. For example, HIV-1 replication is influenced by the host CypA expression level [31]. It has also been reported that the higher cellular CypA content in some cells allows HIV-1 virus replication to occur by compensating for the decreased binding affinity of the P222A Gag mutant for CypA [32]. Poor infectivity of HIV-1 virions, particularly in arrested cells, is caused by disturbed uncoating under the combined action of high CypA levels and capsid mutations [33]. Further, the different CypA levels in HeLa and Jurkat cells may contribute to the CypA-CA binding either stabilizing or destabilizing the HIV-1 capsid, which is associated with the efficiency of HIV-1 infection [34]. In the present study, these phenomena which were observed (Figure 2) demonstrated the relevance of CypA levels to influenza virus infectivity. As previously reported, CypA is cytosolic and found in all tissues in mammals [35]. The different contents of CypA in different species may be related to the infectivity of influenza virus in the host species and CypA may play critical roles as a host restriction factor.

To our knowledge, this is the first report to show that CypA plays an important role in viral replication through accelerating the degradation of a viral protein. Recently, the peptidyl-prolyl cis/trans isomerase Pin1, another member of the cyclophilin family, was reported to stabilize the human T-cell leukemia virus type 1 (HTLV-1) Tax oncoprotein and promote malignant transformation [22]. This suggests that the cyclophilin family may be involved in the regulation of viral replication at different stages by affecting the stability of various viral proteins. Thus, it is of interest to further study the detailed effect of the cyclophilins, with a focus on the proteasome system upon viral replication.

As the major intracellular protein degradation pathway, the UPS also regulates many basic cellular processes. Inhibition of the UPS affects the replication of several viruses, As for HIV-1, TRIM 5α accelerates the degradation of cytosolic capsid protein normally associated with productive HIV-1 entry [36]. In contrast, the cellular TRIM 5α is degraded upon HIV-1 infection [37]. It was also reported that the proteasome inhibitor MG-132 affected influenza virus replication at a post fusion step [7]. This result suggests that the UPS is necessary for the early stage of influenza virus replication, but the detailed mechanism was not identified. In the present study, CypA took part in the UPS to degrade M1 and thereby inhibit the replication of influenza virus at the post-translational level. These results suggest the struggle between virus offense and host defense using the UPS. Further studies are needed to identify the preferential ubiquitinaton site of M1 so that differences among the different M1 protein subtypes can be compared, which also could indicate the possible relationship between different infectivity and numerous subtypes of influenza virus.

## Materials and Methods

### Cell lines, virus, and antibodies

The Madin-Darby Canine Kidney (MDCK) cell line (ATCC CCL-34), human embryonic kidney 293T [38] were maintained in Dulbecco's modified Eagle's medium (GIBCO) supplemented with 10% heat-inactivated fetal bovine serum (GIBCO). Wild-type influenza A virus strain A/WSN/33 (H1N1) was used in these experiments. Rabbit polyclonal antibodies against CypA, CypE, and NP were generated by immunization of 2-month-old female rabbits with 250 µg purified hexahistidine-tagged CypA (His-CypA), His-CypE, or His-NP in Freund's complete adjuvant. The generation of antibodies was boosted three times by immunization with 150 µg of the protein at 2-week intervals. Mouse anti-M1 monoclonal antibody was prepared as described previously [39]. Anti-β-actin (sc-1616-R), anti-lamin B1 was purchased from Santa Cruz Biotechnology and anti-α-tubulin was purchased from Sigma. FITC-conjugated anti-Rabbit IgG and FITC-conjugated anti-mouse IgG were purchased from Zhongshan Golden Bridge Biotechnology (Beijing, China).

### shRNA-based depletion of CypA in 293T cells

The pSUPER RNAi System (Oligoengine, Seattle, WA) was used to generate a viral vector (pSR-CypA) to produce an shRNA to CypA. The oligos used for the shRNA were 5′GATCCCCGGGTTCCTGCTTTCACAGATTCAAGAGATCTGTGAAAGCAGGAACCCTTTTTGGAAA-3′ and 5′-AGCTTTTCCAAAAAGGGTTCCTGCTTTCACAGATCTCTTGAATCTGTGAAAGCAGGAACCCGGG-3′ [27]. The oligo duplex was cloned into the *Bgl*II/*Hin*dIII sites of pSRgfp/neo. A stable cell line expressing the shRNA to CypA was generated using a retroviral spin infection. Retroviral supernatant was generated from 293T cells transfected with the pSR-shCypA with the pCL-Eco packaging construct and pCL-VSVG. The 293T cells were grown to sub-confluence in a 12-well dish. Cells and viral supernatant were then centrifuged at 2,500 rpm for 2 h at 32°C with 8 µg/ml polybrene. Several clonal populations of GFP-expressing cells were obtained and measured for CypA depletion. To measure CypA depletion, a short-length (<1 kb) cDNA library was generated from total RNA using an Aurum total RNA mini kit (Bio-RAD, Hercules, CA) and an iScript cDNA Synthesis kit (Bio-Rad, Hercules, CA). CypA primers, position 1–20 and position 147–166, were used for quantitative real-time PCR. Primers targeting the CypA intron, β-actin, and GAPDH were used as controls. Primer sequences are available upon request. SYBR green supermix, the MyiQ single-color PCR detection system, and optical system software (Bio-Rad) were used for real-time PCR [40]

### Indirect immunofluorescence

The 293T/CypA+ and 293T/CypA− cells, growing on glass cover slips, were infected with WSN at an MOI of 0.1. The cells were then washed with PBS three times, fixed in 4% paraformaldehyde for 30 min at room temperature, permeabilized with 0.5% triton X-100 in PBS (PBST) for 20 min, and stained with appropriate antibodies. Cell nuclei were stained with 5 µg/ml DAPI (Sigma) in PBS. Following staining, cover slips were analyzed using an LSCMFV500 confocal microscope.

### Plaque assay

MDCK cell monolayers (1.5×10^6^ cells at a confluency of 100% in a 12-well chamber) were washed with PBS and infected with different dilutions of virus for 1 h at 37°C. The virus inoculums were removed by washing with PBS. Cell monolayers were then overlaid with agar overlay medium (DMEM supplemented with 1% low melting point agarose and 1 µg/ml TPCK-treated trypsin) and incubated at 37°C. Visible plaques were counted at 4 days post-infection, and the virus titers were determined. All data are expressed as the mean of three independent experiments.

### Virus infection and infectivity assay

Equal numbers of 293T/CypA+ and 293T/CypA− cells were grown on cover slips in a 24-well chamber and infected with A/WSN/33 (MOI = 1). At 4 h post-infection, the cells were fixed, and analyzed by indirect immunofluorescence using anti-NP antibody and FITC-conjugated anti-rabbit IgG antibody.

### RNA extraction and cDNA synthesis

Equal numbers of 293T/CypA+ and 293T/CypA− cells were cultivated and infected with WSN (MOI = 0.1). Total RNA was extracted at 0, 2, 4, 6, and 8 h post-infection using Trizol reagent (Invitrogen). The purified RNA samples were treated with DNase I (TaKaRa) for 30 min at 37°C. Total RNA (1.5 µg) was reverse transcribed by AMV reverse transcriptase (Promega) according to the protocol provided by the manufacturer and 10 pmol of cRNA-specific primer (primer: 5′-AGTAGAAACAAGG-3′), 10 pmol of vRNA-specific primer (primer 1: 5′-AGCGAAAGCAGG-3′; primer 2: 5′-AGCAAAAGCAGG-3′) [41], or 10 pmol of oligo-dT primer. AMV reverse transcriptase exonuclease activity was then heat inactivated at 95°C for 5 min. Negative controls (amplifications in the absence of RNA or primers) were included in parallel to ascertain absence of contamination by template nucleic acids and the efficiency in RT inactivation. cDNA was stored at −80°C.

### Real-time PCR analysis

The analysis of the relative M1 gene expression was performed using Corbett 6200 and PCR primers: M1 forward (5′-TCTGATCCTCTCGTCATTGCAGCAA-3′) and M1 reverse (5′-AATGACCATCGTCAACATCCACAGC-3′) [3]. GAPDH served as an internal control using PCR primers: GAPDH forward (5′-GGTGGTCTCCTCTGACTTCAACA-3′) and GAPDH reverse (5′-GTTGCTGTAGCCAAATTCGTTGT-3′), as described in [42]. The cycling conditions comprised an initial denaturation step of 30 s at 95°C, followed by 40 two-step cycles (95°C for 5 s and 60°C for 30 s). Dissociation curve analysis was performed after each assay to ensure specific target detection. All data and standard deviations were determined from three independent experiments.

### Nuclear and cytoplasmic mRNA quantification by real-time PCR

Nuclear and cytoplasmic RNA was fractionated from cells as described in [43]. Briefly, the cells were lysed in RSB (10 mM Tris, pH 7.4, 10 mM NaCl, 3 mM MgCl_2_) containing 0.5% Nonidet P-40, 10% glycerol, and 100 units/ml rRNasin (Promega). Nuclei were further washed with 1% Tween-40 and 0.5% sodium deoxycholate, and RNA from both cytoplasmic and nuclear fractions was purified using Trizol (Invitrogen). The purified RNA samples were treated with DNase I (TaKaRa) for 30 min at 37°C. One microgram of total RNA was used for cDNA synthesis with the oligo(dT)18 primer, followed by real-time RT-PCR quantification. Negative controls (amplifications in the absence of RNA or primers) were included in parallel to ascertain absence of contamination by template nucleic acids and the efficiency in RT inactivation. The resulting relative M1 and NP mRNA levels were normalized by the geometric mean of the housekeeping gene (GAPDH).

### Protein degradation assay

Both 293T/CypA+ and 293T/CypA− cell lines were transfected with pcDNA3-FLAG-M1 or pcDNA3-FLAG-NP plasmids. CHX (100 µg/ml) was added to the medium 24 h post-transfection, and the cells were harvested at different time points. Western blots were probed with anti-β-actin, anti-M1, anti-NP, and anti-CypA antibodies. MG-132 and NH_4_Cl were used at the same time as CHX, and the cells were harvested 8 h after treatment.

### In vivo ubiquitination assays

293T/CypA− cells were transfected with expression constructs for Myc-CypA, FLAG-tagged full-length M1, and His-tagged ubiquitin. Thirty-two hours post-transfection, the proteasome inhibitor MG-132 (dissolved in DMSO) was added to the cells for 4 h at a final concentration of 10 µM. Cells from each plate were collected into two aliquots. One aliquot (10%) was used for conventional western blotting to confirm expression and degradation of the transfected proteins. The remaining cells (90%) were used for purification of the His_6_-tagged proteins by Ni^2+^-nitrilotriacetic acid (NTA) beads. The cell pellet was lysed in buffer A (6 M guanidinium-HCl, 0.1 M Na_2_HPO_4_/NaH_2_PO_4_, 0.01 M Tris-Cl [pH 8.0], 5 mM imidazole, and 10 mM β-mercaptoethanol) and incubated with Ni^2+^-NTA beads (Qiagen) for 4 h at room temperature. The beads were then washed with buffer A, buffer B (8 M urea, 0.1 M Na_2_PO_4_/NaH_2_PO_4_, 0.01 M Tris-Cl [pH 8.0], and 10 mM β-mercaptoethanol), and buffer C (8 M urea, 0.1 M Na_2_PO_4_/NaH_2_PO_4_, 0.01 M Tris-Cl [pH 6.3], and 10 mM β-mercaptoethanol), and bound proteins were eluted with buffer D (200 mM imidazole, 0.15 M Tris-Cl [pH 6.7], 30% glycerol, 0.72 M β-mercaptoethanol, and 5% SDS). The eluted proteins were analyzed by western blotting using an anti-FLAG monoclonal antibody.
